# Supplementary material for: Recent Advances and Future Directions in Alzheimer’s Disease Genetic Research
Source: Int J Mol Sci. 2025 Aug 13;26(16):7819. doi: 10.3390/ijms26167819 (PMC12386822; doi:10.3390/ijms26167819)
Supplement: Supplementary file 1 [file ijms-26-07819-s001.zip › ijms-3739791-supplementary.pdf]

Supplementary Table S1. Risk and protective variants for Alzheimer's disease in *CR1, BIN1, TREM2, ZCWPW1, CLU, MS4A4, PICALM, SORL1, ADAM10, ABCA7, APOE, CD33* and *APP*.

OR-odds ratio

Gene	SNP	A1	A2	FRQ	gnomAD global allele frequency	OR (95% CI))	*p*-value	Reference
CR1	rs6656401	G	A	0.197	0.862182	1.18 (1.14–1.22)	5.7E−24	[1]
CR1	rs679515	C	T	0.188	0.862653	1.13 (1.11–1.15)	7.2E−46	[2]
CR1	rs2093760	G	A	0.192	0.73385	1.02	1.1E-18	[3]
CR1	rs4844610	C	A	0.187	0.862288	1.17 (1.13–1.21)	3.6E−24	[4]
BIN1	rs4663105	A	C	0.411	0.462149	1.03	3.38E-44	[3]
BIN1	rs6733839	C	T	0.409	0.395051	1.22 1.18–1.25)	6.9E−44	[1]
BIN1	rs6733839	C	T	0.389	0.395051	1.17 (1.16–1.19)	6.1E−118	[2]
TREM2	rs143332484	C	T	0.013	0.00859818	1.41 (1.32–1.50)	2.8E−25	[2]
TREM2	rs75932628	C	T	0.003	0.00152857	2.39 (2.09–2.73)	2.5E−37	[2]
ZCWPW1	rs1476679	T	C	0.287	0.774949	0.91 (0.89–0.94)	5.6E−10	[1]
CLU	rs4236673	G	A	0.378	0.700579	0.98	2.61E-19	[3]
CLU	rs11787077	C	T	0.392	0.573665	0.91 (0.90–0.92)	1.7E−44	[2]
CLU	rs1532278	C	T	0.39	0.680899	0.988	1.57E−22	[5]
CLU	rs9331896	T	C	0.379	0.569712	0.86 (0.84–0.89)	2.8E−25	[1]
MS4A4A	rs2081545	C	A	0.382	0.290974	0.982	1.55E-15	[3]
MS4A4A	rs1582763	G	A	0.38	0.260865	0.985	3.40E−33	[3]
MS4A4A	rs1582763	G	A	0.371	0.260865	0.91 (0.90-0.92)	3.7E−42	[2]
PICALM	rs867611	A	G	0.317	0.730376	0.98	2.19E-18	[3]
PICALM	rs561655	A	G	0.35	0.695311	0.986	1.57E−22	[5]
PICALM	rs10792832	G	A	0.358	0.700249	0.87 (0.85–0.89)	9.3E−26	[1]
SORL1	rs74685827	T	G	0.019	0.0176489	1.19 (1.13-1.25)	2.8E−11	[2]
SORL1	rs11218343	T	C	0.039	0.0645089	0.77 (0.72–0.82)	9.7E−15	[1]

SORL1	rs11218343	T	C	0.039	0.0645089	0.84 (0.81–0.87)	1.4E−2	[2]
ADAM10	rs442495	T	C	0.354	0.487648	0.986	1.31E-09	[3]
ADAM10	rs593742	A	G	0.295	0.336907	0.93 (0.91-0.95)	6.8E−9	[4]

References

1. Lambert, J.C.; Ibrahim-Verbaas, C.A.; Harold, D.; Naj, A.C.; Sims, R.; Bellenguez, C.; DeStafano, A.L.; Bis, J.C.; Beecham, G.W.; Grenier-Boley, B.; et al. Meta-analysis of 74,046 individuals identifies 11 new susceptibility loci for Alzheimer's disease. *Nature genetics* **2013**, *45*, 1452-1458, doi:10.1038/ng.2802.
2. Bellenguez, C.; Küçükali, F.; Jansen, I.E.; Kleineidam, L.; Moreno-Grau, S.; Amin, N.; Naj, A.C.; Campos-Martin, R.; Grenier-Boley, B.; Andrade, V.; et al. New insights into the genetic etiology of Alzheimer's disease and related dementias. *Nature genetics* **2022**, *54*, 412-436, doi:10.1038/s41588-022-01024-z.
3. Jansen, I.E.; Savage, J.E.; Watanabe, K.; Bryois, J.; Williams, D.M.; Steinberg, S.; Sealock, J.; Karlsson, I.K.; Hägg, S.; Athanasiu, L.; et al. Genome-wide meta-analysis identifies new loci and functional pathways influencing Alzheimer's disease risk. *Nature genetics* **2019**, *51*, 404-413, doi:10.1038/s41588-018-0311-9.
4. Kunkle, B.W.; Grenier-Boley, B.; Sims, R.; Bis, J.C.; Damotte, V.; Naj, A.C.; Boland, A.; Vronskaya, M.; van der Lee, S.J.; Amlie-Wolf, A.; et al. Genetic meta-analysis of diagnosed Alzheimer's disease identifies new risk loci and implicates Aβ, tau, immunity and lipid processing. *Nature genetics* **2019**, *51*, 414-430, doi:10.1038/s41588-019-0358-2.
5. Wightman, D.P.; Jansen, I.E.; Savage, J.E.; Shadrin, A.A.; Bahrami, S.; Holland, D.; Rongve, A.; Børte, S.; Winsvold, B.S.; Drange, O.K.; et al. A genome-wide association study with 1,126,563 individuals identifies new risk loci for Alzheimer's disease. *Nature genetics* **2021**, *53*, 1276-1282, doi:10.1038/s41588-021-00921-z.

Supplementary Table S2. Most cited clinical trials regarding Alzheimer's disease treatments targeting the amyloidogenic, tau-related and neuroinflammatory pathway.

**Drug**	**Developer**	**Mechanism of Action**	**Stage**	**NCT Number**
Aducanumab	Biogen/Eisai	Aβ-specific monoclonal antibody	Phase III (approved/controversial)	NCT02484547
Lecanemab (Leqembi)	Eisai/Biogen	Aβ-specific monoclonal antibody	Phase III (approved)	NCT03887455 (CLARITY-AD)
Donanemab (TRAILBLAZER-AD 2)	Eli Lilly	Aβ-specific monoclonal antibody	Phase III	NCT04437511
Gantenerumab (RO4909832)	Roche/Genentech	Aβ-specific monoclonal antibody	Phase III (terminated)	NCT03443973
Solanezumab (LY2062430)	Eli Lilly	Aβ-specific monoclonal antibody	Phase III (terminated/discontinued)	NCT02760602/A4 / DIAN-TU
Remternetug	Eli Lilly	Anti-pyroglutamated Aβ antibody	Phase III (TRAILRUNNER-ALZ1)	NCT05463731
ALZ-801 (tramiprosate prodrug)	Alzheon	Small-molecule Aβ oligomer inhibitor	Phase III (APOLLOE4)	NCT04770220
Simufilam (PTI-125)	Cassava Sciences	Filamin-A stabilizer, reduces Aβ–receptor interactions	Phase III (RETHINK-ALZ / REFOCUS-ALZ)	NCT0499448, NCT05026177
PBT2	Prana Biotech	Metal-protein attenuating compound (modulates Aβ & cognition)	Phase II	PBT2-204/IMAGINE (no NCT ref)
Verubecestat (MK-8931)	Merck & Co.	BACE-1 inhibitor	Phase III (terminated)	NCT01953601/NCT02036280
Semagacestat (LY-450139)	Eli Lilly / Elan	γ-secretase inhibitor	Phase III (terminated)	NCT00695573 (IDENTITY-1/2)
Xanamem (AC-1202)	Actinogen Medical	Cortisol synthesis inhibitor (metabolic/neuroprotective)	Phase II/III	NCT01741194 or early trials
AGB101 (levetiracetam)	Various	SV2A modulator	Phase II/III (recruiting)	NCT03486938

AXS-05	Axsome Therapeutics	Sigma-1 receptor agonist, NMDA antagonist & NE/DA reuptake inhibitor	Phase III (agitation symptoms)	NCT03226522
COR388	Cortexyme	Bacterial protease inhibitor	Phase II/III (neuroinflammatory)	NCT03823404
Azeliragon	vTv Therapeutics / Pfizer	RAGE antagonist (microglial activation)	Phase II/III (terminated)	NCT02916056/others
Masitinib	AB Science	Tyrosine kinase inhibitor (neuroinflammatory; mast cell regulator)	Phase III	NCT05185529
Remnet / AR1001	AriBio	Investigational (amyloid-targeting)	Phase III	various
Aducanumab recruitments	Biogen	Aβ antibody	Phase III	NCT01397539, NCT02484547
Lecanemab enrollment	Eisai/Biogen	Aβ antibody	Phase III	NCT03887455
